# Material property alterations for phenotypes of heart failure with preserved ejection fraction: A numerical study of subject-specific porcine models

**DOI:** 10.3389/fbioe.2022.1032034

**Published:** 2022-10-14

**Authors:** Jonathan Weissmann, Christopher J. Charles, A. Mark Richards, Choon Hwai Yap, Gil Marom

**Affiliations:** ^1^ Department of Biomedical Engineering, Tel Aviv University, Tel Aviv, Israel; ^2^ Department of Surgery, Yong Loo Lin School of Medicine, National University of Singapore, Singapore, Singapore; ^3^ Cardiovascular Research Institute, National University of Singapore, Singapore, Singapore; ^4^ Christchurch Heart Institute, Department of Medicine, University of Otago, Christchurch, New Zealand; ^5^ Department of Bioengineering, Imperial College London, London, United Kingdom; ^6^ School of Mechanical Engineering, Tel Aviv University, Tel Aviv, Israel

**Keywords:** heart failure with preserved ejection fraction, phenotypes, finite element analysis, computational modelling, material properties, animal modeling

## Abstract

A substantial proportion of heart failure patients have a preserved left ventricular (LV) ejection fraction (HFpEF). This condition carries a high burden of morbidity and mortality and has limited therapeutic options. left ventricular pressure overload leads to an increase in myocardial collagen content, causing left ventricular stiffening that contributes to the development of heart failure patients have a preserved left ventricular ejection fraction. Although several heart failure patients have a preserved left ventricular ejection fraction models have been developed in recent years to aid the investigation of mechanical alterations, none has investigated different phenotypes of the disease and evaluated the alterations in material properties. In this study, two similar healthy swine were subjected to progressive and prolonged pressure overload to induce diastolic heart failure characteristics, providing a preclinical model of heart failure patients have a preserved left ventricular ejection fraction. Cardiac magnetic resonance imaging (cMRI) scans and intracardiac pressures were recorded before and after induction. In both healthy and disease states, a corresponding finite element (FE) cardiac model was developed *via* mesh morphing of the Living Heart Porcine model. The material properties were derived by calibrating to its passive and active behavior. The change in the passive behavior was predominantly isotropic when comparing the geometries before and after induction. Myocardial thickening allowed for a steady transition in the passive properties while maintaining tissue incompressibility. This study highlights the importance of hypertrophy as an initial compensatory response and might also pave the way for assessing disease severity.

## Introduction

Heart failure (HF) is a leading cause of morbidity and mortality (Roger, 2021). HF with preserved ejection fraction (HFpEF) is characterized by an ejection fraction above 50% and increased left ventricular (LV) filling pressure (Heidenreich et al., 2022). It is estimated to affect a considerable proportion of HF patients (Clark and Velazquez, 2020). In the United States alone, the prevalence of HF is estimated to be 6 million, which is approximately 1.8% of the total United States population (Roger, 2021). HFpEF is associated with increased morbidity and mortality, including a 35% 2-year rate of HF hospitalization and a 14% 2-year mortality (Dunlay et al., 2017), even in asymptomatic patients (Kane et al., 2011). The prevalence rate continues to rise significantly owing to the aging of the population and the increasing prevalence of comorbidities such as hypertension, obesity, and diabetes (AlJaroudi et al., 2012; Shah and Solomon, 2012; Kitzman and Shah, 2016; Virani et al., 2020). Despite the large number of patients, few pharmacological or device-driven therapies have been shown to improve clinical outcomes or lower mortality rates (Bhuiyan and Maurer, 2011; Wintrich et al., 2020; Anker et al., 2021).

A key contributing etiology of HFpEF is LV stiffening due to myocardial remodeling. Progressive and prolonged LV pressure overload leads to an increase in myocardial collagen content, causing both regional and global LV stiffening as well as elevated LV filling pressures (Borbély et al., 2005; Zile et al., 2015). However, this stiffening mechanism is still not fully understood, and the variety of phenotypes makes an accurate assessment difficult (Shah and Solomon, 2012; Sengupta and Marwick, 2018; Shah et al., 2020; Roh et al., 2022).

Numerical analysis enables non-invasive mechanical assessment of the cardiovascular system (Chandran, 2010; Niederer et al., 2011; Votta et al., 2013; Sahli Costabal et al., 2018; Wisneski et al., 2020; Cutugno et al., 2021). In recent years, models have been developed to investigate the impact of HFpEF on heart function (Poh et al., 2012; Adeniran et al., 2015; Dabiri et al., 2018; Escher et al., 2020). Global tissue stiffness has been examined using finite element (FE) modeling by comparing the mechanical properties of an HFpEF cardiac ventricle to a healthy heart of a different subject (Sack et al., 2018). Torres et al. (2020) have recently investigated LV stiffening in response to progressive pressure overload. A predictor variable for LV stiffness was derived from computed stress magnitudes in the circumferential and longitudinal directions. It was based on clinical echocardiogram strain measurements in selective heart regions and the adaptation of an ellipsoidal model. Although tissue stiffening has been corroborated by these models, a more comprehensive description of the material properties is required to establish a better pathophysiological understanding.

In this study, we aim to compare material properties before and after the elevation of the LV filling pressure. Four FE models were developed for healthy and HFpEF scenarios based on an animal study, where swine were recorded before and after HFpEF induction. This study is the first to compare healthy and HFpEF conditions of the same subject by calibrating their material properties. The same technique can be applied to a larger study population, to quantify the changes in material properties in HFpEF and aid in the classification of disease variants.

## Materials and methods

### Animal experiment

Yorkshire Landrace pigs at the age of 3–4 months were subjected to progressive and prolonged pressure overload using an aortic inflatable cuff over a 5-week period to induce diastolic HF characteristics (Charles et al., 2020). Cardiac magnetic resonance imaging (cMRI) scans and LV pressures were recorded on days 1 and 42, pre- and post-cuff placement, representing healthy and disease conditions, respectively. A subset of two normal pigs with similar body weight (19.9 and 20.5 kg), surface area (0.74 and 0.77 m^2^), and cardiac characteristics (Table 1) were chosen from the full cohort. During the experiment, the two pig models demonstrated good development of HFpEF characteristics, including LV hypertrophy and preserved ejection fraction. The study was conducted as part of the Asian neTwork for Translational Research and Cardiovascular Trials (ATTRaCT) program and was authorized by the Institutional Animal Care and Use Committee (IACUC) of the National University of Singapore (Protocol R15-0090).

**TABLE 1:** Pressure and volume magnitudes of the healthy configuration before the HF induction.

	End-systole		End-diastole		Maximum pressure (mmHg)	Stroke volume (ml)	Ejection fraction (%)
	Volume (ml)	Pressure (mmHg)	Volume (ml)	Pressure (mmHg)			
*Case 1*	22	81	55	12	85	34	61
*Case 2*	20	80	56	13	84	36	64

### Geometrical modeling

For each pig model, the geometries of normal and HFpEF anatomies were reconstructed based on recordings before and after HF induction. The recordings were segmented at end diastole and were followed by smoothing with volume preservation (Weissmann et al., 2021). The living heart porcine model (LHPM; Simulia, Dassault Systèmes, Providence, RI, United States) (Baillargeon et al., 2014, 2017; SIMULIA, 2016) was morphed based on the reconstructed geometries, using Abaqus solver (Simulia, Dassault Systèmes), to generate multiple FE models. A detailed description of the algorithm can be found in our published work (Weissmann et al., 2021).

For each pig, the LV wall thickness was measured before and after the induction of HFpEF to evaluate the muscular growth due to hypertrophy. Short-axis slices at end-diastole were analyzed and the LV mass (LVM) was calculated by subtracting endocardial from epicardial volume. The value was normalized to body surface area (LVMI), which was calculated using the Mosteller formula (Mosteller, 1987).

### Material properties calibration

The material characteristics of the LV tissue include passive and active properties embedded in the LHPM (Simulia, 2016). The passive behavior was defined according to the Holzapfel and Ogden (2009) constitutive model. The strain energy function is comprised of isochoric $\left(\psi_{iso}\right)$ and volumetric $\left(\psi_{vol}\right)$ components as follows:

$$\psi_{iso}=\frac{a}{2b}e^{b\left(I_{1}-3\right)}+\sum_{i=f,s}\frac{a_{i}}{{2b}_{i}}\left\{e^{{b_{i}\left(I_{4i}-1\right)}^{2}}-1\right\}+\frac{a_{fs}}{{2b}_{fs}}\left\{e^{{b_{fs}\left(I_{8fs}\right)}^{2}}-1\right\}$$

$$\psi_{vol}=\frac{1}{D}\left(\frac{J^{2}-1}{2}-\ln\left(J\right)\right)$$

where the tissue stiffness is determined in the isotropic direction (*a*, *b*), fiber direction (*a*_*f*,_ *b*_*f*_), sheet direction (*a*_*s*,_ *b*_*s*_), and the connection between the fibers and sheet (*a*_*fs*,_ *b*_*fs*_). $I_{1}:=tr\left(C\right)$ , $I_{4i}:=C:\left(f_{0}⊗f_{0}\right)$ , and $I_{8fs}:=C:sym\left(f_{0}{⊗s}_{0}\right)$ are invariants, *C* is the right Cauchy-Green tensor, and *f*_0_ and *s*_0_ are vectors that define the fiber and sheet directions, respectively. Sym () operator represents symmetrical disposition with respect to the axes of deformation (Merodio and Ogden, 2006), *J* is the third deformation gradient invariant, and *D* = 2/*K* where *D* is the incompressibility parameter and *K* is the bulk modulus.

The coefficients of the isochoric function were determined using the analytic pressure-volume curve suggested by Klotz et al. (2006). The curve was customized for each case with the end diastolic pressure (EDP) and end diastolic volume (EDV) obtained from clinical measurements. Further details of the configuration of the “Klotz” curve are provided in the Supplementary Material.

A genetic algorithm was implemented to obtain an optimal set of passive material parameters matching the Klotz curve while accounting for local and global minima. An initial generation was chosen randomly, and for each parameter, specified bounds were chosen to allow a wide range of physiological values (Göktepe et al., 2011; Wang et al., 2013; Nikou et al., 2015) (Table 2). The algorithm used default settings for selection, recombination, and mutation (Pohlheim and Marenbaeh, 1996). Each set of parameters was ranked by a fitness function, which computed an overall distance between the desired Klotz curve and an FEA-generated pressure-volume curve. The next generation was created by applying recombination and mutation attributes to the sets of parameters with the lowest scores. Iterative selection was performed for three generations until a score within 2.5% of the target value was found. This process was implemented using an in-house *Python* script, which generated an Abaqus input file for each set of parameters, executed the solver, and analyzed the FEA results.

**TABLE 2:** The passive parameter bounds.

Parameter	Lower bound	Upper bound
*a* (MPa)	$1.0\times 10^{-5}$	$3.5\times 10^{-3}$
*b*	1	25
*a*_*f*_ (MPa)	$5.5\times 10^{-5}$	$5.0\times 10^{-3}$
*b*_*f*_	5	90
*a*_*s*_ (MPa)	$1.0\times 10^{-5}$	$1.0\times 10^{-3}$
*b*_*s*_	5.5	50.5
*a*_*fs*_ (MPa)	$4.5\times 10^{-5}$	$2.0\times 10^{-4}$
*b*_*fs*_	2.25	10

For each model, a second calibration of the HFpEF conditions was performed to quantify potential changes in the passive behavior by eliminating the influence of geometrical alterations. The healthy FE models, prior to the induction, were compared to the Klotz curves, using volume and pressure measurements taken after the induction of HFpEF. The algorithm ran under identical conditions and predefined boundaries as in the first calibration. To enable more degrees of freedom, as an alternative to hypertrophy, the incompressibility parameter *D* was also included in the calibration. The first calibration values were utilized as an initial guess in the second calibration to obtain minimal alterations relative to the healthy state.

To synchronize the pressure and volume magnitudes during heart contraction, an alteration in contractility was facilitated by the active sarcomere tension (Guccione and McCulloch, 1993; Genet et al., 2014; Sack et al., 2016). More information can be found in the supplementary material section.

### Circulatory system

**FIGURE 1:**
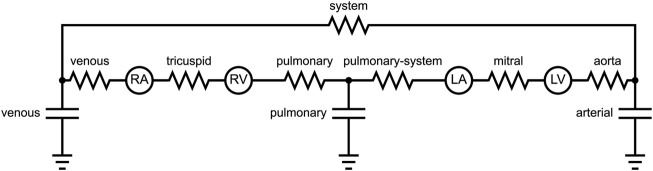
Electrical representation of the blood flow model.

The embedded blood flow model in the LHPM uses a lumped parameter approach. Figure 1 shows a schematic illustration of the blood flow model, with resistors representing flow resistances and capacitors representing structural compliance. The blood flow was modeled by inducing fluid exchange between the various heart chambers. The capacitors are mechanically represented by reservoirs and their pressure-volume response is controlled by springs. For each model, the venous compliance and the aortic valve, systemic and venous resistances were adjusted to obtain pressure–volume loops similar to clinical measurements. The HFpEF lumped settings and the corresponding healthy ones differed only in their resistance, such that the aortic and systemic resistances were increased in our HFpEF models, representing the aortic cuffing that induced HFpEF in the animal. To maintain proper venous return in our models, the venous resistance was also slightly lowered. The lumped parameters are listed in Supplementary Table S1.

## Results

### Model geometry

**FIGURE 2:**
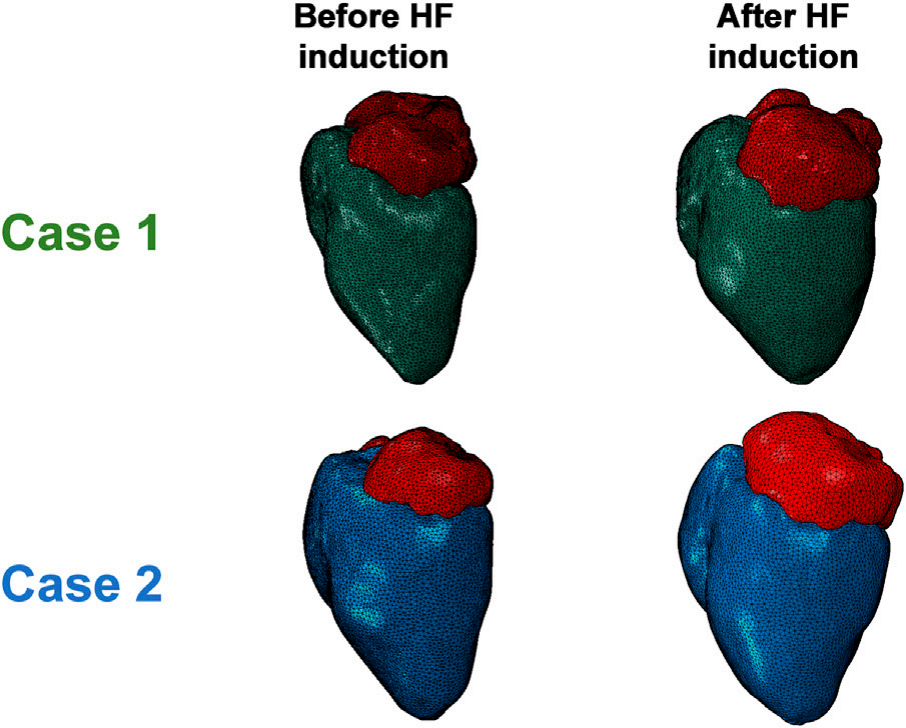
Comparison of the morphed FE models of the two cases’ anatomies before and after HFpEF induction.

**FIGURE 3:**
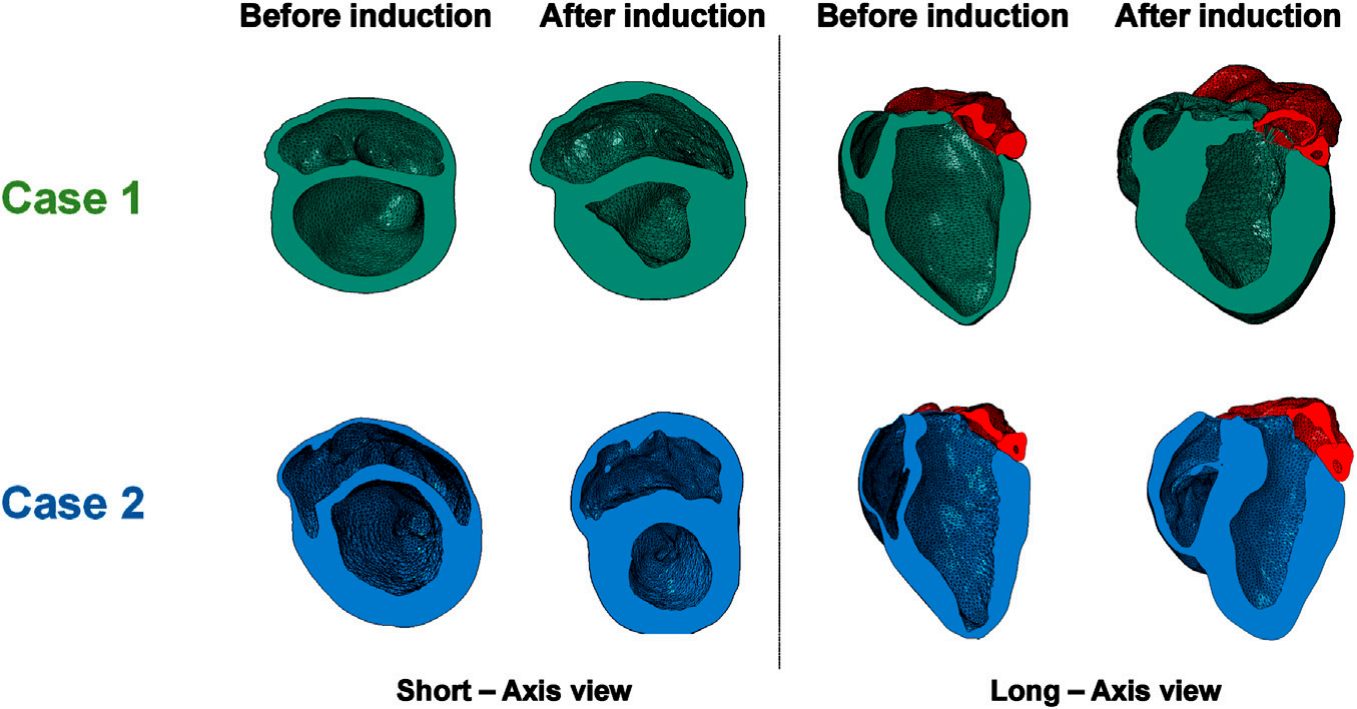
Long and short axis views of the two cases before and after HFpEF induction.

**FIGURE 4:**
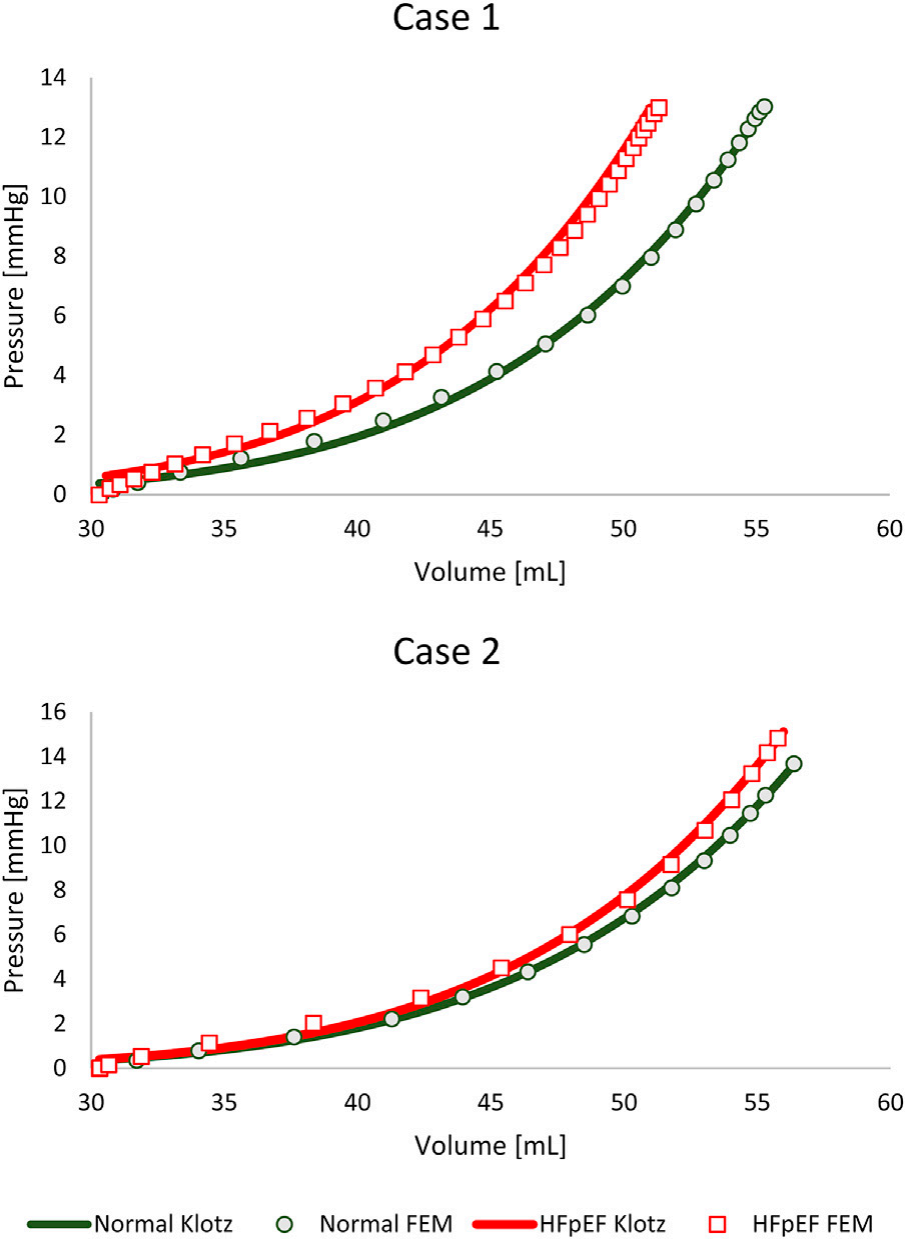
The passive filling FE results and the corresponding “Klotz” curves for the four models.

A visualization of the FE models following cMRI segmentation and mesh morphing is shown in Figure 2. The LHPM was used as a baseline mesh, and was morphed to generate cardiac models matching those of our animal models, both before and after induction of HFpEF. A previously developed mesh-morphing algorithm was used for this, which can accurately match imaged wall and septal thicknesses, and has the advantage of preserving fine structural details captured in the baseline mesh (Crick et al., 1998; Weissmann et al., 2021). For each model, long- and short-axis cross sections in the initial healthy configuration and the post induction HF configuration are presented in Figure 3. The LV mass value was determined for each case and is shown in Table 3. Albeit similar physical properties at normal condition, the mass properties have changed after induction, as the hypertrophy in Case 1 was substantially larger, predominantly if normalized to the body surface area. (Figure 4; Table 3).

**TABLE 3:** Left ventricular mass properties before and after induction.

	Case 1			Case 2		
	Normal	HFpEF	Growth (%)	Normal	HFpEF	Growth (%)
*LVM* (*g*)	49	132	269	49	122	250
*LVMI* (gm2)	66	136	206	63	120	190

### Material parameter estimation

**FIGURE 5:**
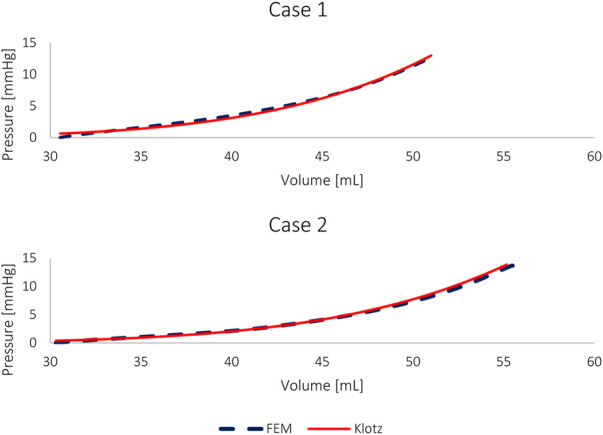
The pressure-volume curves of the FE models and the corresponding Klotz curves of HFpEF models without geometric alterations.

The material parameters (*a*, *b*, *a*_*f*_, *b*_*f*_, *a*_*s*_, *b*_*s*_, *a*_*f*s_, *b*_*fs*_) were calibrated for the subject-specific anatomies and produced appropriate results for all models, as previously reported in the literature (Göktepe et al., 2011; Wang et al., 2013; Nikou et al., 2015; Genet et al., 2016; Dabiri et al., 2018; Peirlinck et al., 2019; Heidari et al., 2022). Table 4 lists the values of the eight calibrated passive material parameters for the two cases. The FE passive filling curves were plotted against the corresponding analytical Klotz curves to graphically highlight the optimization efficiency (Figure 5). *R* ^2^ scores were calculated for quantitative fitting evaluation, with *R* ^2^ of 0.999 and 0.997 for the healthy and unhealthy configurations, respectively, in both models.

**TABLE 4:** The passive material properties for each cardiac model before and after the induction of HFpEF.

	Case 1		Case 2	
	Normal	HFpEF	Normal	HFpEF
*a* (MPa)	$8.69\times 10^{-4}$	$2.12\times 10^{-4}$	$1.43\times 10^{-4}$	$1.48\times 10^{-3}$
*b*	$1.51\times 10^{+1}$	$1.96\times 10^{+1}$	4.28	$1.23\times 10^{+1}$
*a*_*f*_ (MPa)	$2.26\times 10^{-3}$	$3.83\times 10^{-3}$	$4.86\times 10^{-3}$	$4.86\times 10^{-3}$
*b*_*f*_	$4.33\times 10^{+1}$	$8.42\times 10^{+1}$	$8.63\times 10^{+1}$	$8.63\times 10^{+1}$
*a*_*s*_ (MPa)	$8.02\times 10^{-4}$	$8.89\times 10^{-4}$	$8.61\times 10^{-4}$	$8.61\times 10^{-4}$
*b*_*s*_	$1.20\times 10^{+1}$	$4.42\times 10^{+1}$	$3.39\times 10^{+1}$	$3.39\times 10^{+1}$
*a*_*fs*_ (MPa)	$1.63\times 10^{-4}$	$1.22\times 10^{-4}$	$8.50\times 10^{-5}$	$1.72\times 10^{-4}$
*b*_*fs*_	9.39	8.50	9.43	5.51

For comparison, the material properties were also calibrated against the HFpEF condition using the healthy anatomy, i.e., without introducing geometric alteration related to hypertrophy. Figure 2 shows this calibration with an identical “Klotz” curve as in Figure 5. The *R* ^2^ scores for a HFpEF condition with normal geometry were 0.998 and 0.999 for Cases 1 and 2, respectively. The differences between the passive properties of normal and HFpEF without anatomical alterations are shown in Table 5. From the results, the major alteration was observed in a single parameter, while the change in the other parameters was minimal. When the calibration accounted for hypertrophic conditions (Table 4), change was observed in all parameters.

**TABLE 5:** The passive material properties for each cardiac model, for normal condition and HFpEF condition while the normal geometry was maintained.

	Case 1		Case 2	
	Normal	HFpEF-Normal Geometry	Normal	HFpEF - Normal Geometry
*a* (MPa)	$8.69\times 10^{-4}$	$8.69\times 10^{-4}$	$1.43\times 10^{-4}$	$1.43\times 10^{-4}$
*b*	$1.51\times 10^{+1}$	$2.01\times 10^{+1}$	4.28	8.28
*a*_*f*_ (MPa)	$2.26\times 10^{-3}$	$2.26\times 10^{-3}$	$4.86\times 10^{-3}$	$4.86\times 10^{-3}$
*b*_*f*_	$4.33\times 10^{+1}$	$4.33\times 10^{+1}$	$8.63\times 10^{+1}$	$8.63\times 10^{+1}$
*a*_*s*_ (MPa)	$8.02\times 10^{-4}$	$8.02\times 10^{-4}$	$8.61\times 10^{-4}$	$8.61\times 10^{-4}$
*b*_*s*_	$1.20\times 10^{+1}$	$1.20\times 10^{+1}$	$3.39\times 10^{+1}$	$3.39\times 10^{+1}$
*a*_*fs*_ (MPa)	$1.63\times 10^{-4}$	$1.63\times 10^{-4}$	$8.50\times 10^{-5}$	$8.50\times 10^{-5}$
*b*_*fs*_	9.39	9.39	9.43	9.43

### Cardiac cycle simulation

**FIGURE 6:**
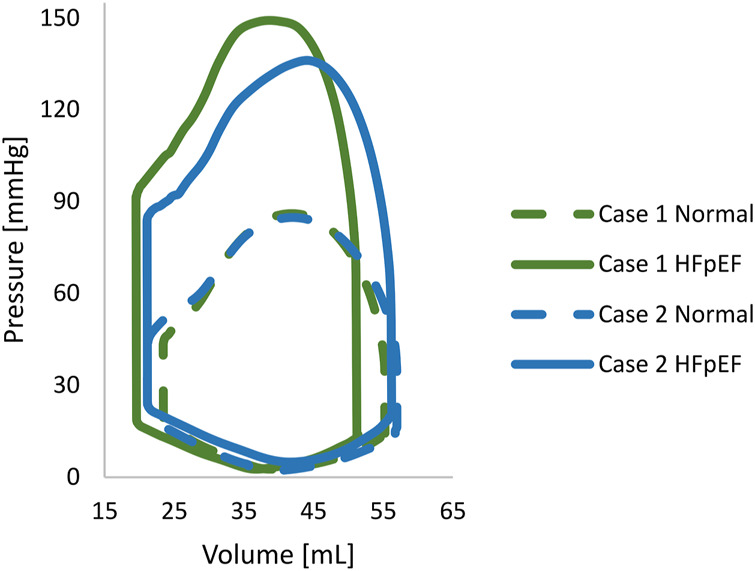
The pressure-volume loop of each porcine normal. The normal and HFpEF configurations are denoted by continuous and dashed lines, respectively.

The sixth cardiac cycle analysis was analyzed to ensure fully converged results (Sack et al., 2018) and stable periodic solutions with closed pressure-volume loops (Figure 6). The computed pressure was within the clinical range for both models before and after the induction, with maximal pressures of 85 mmHg for normal configurations and 150 mmHg for HFpEF conditions.

**FIGURE 7:**
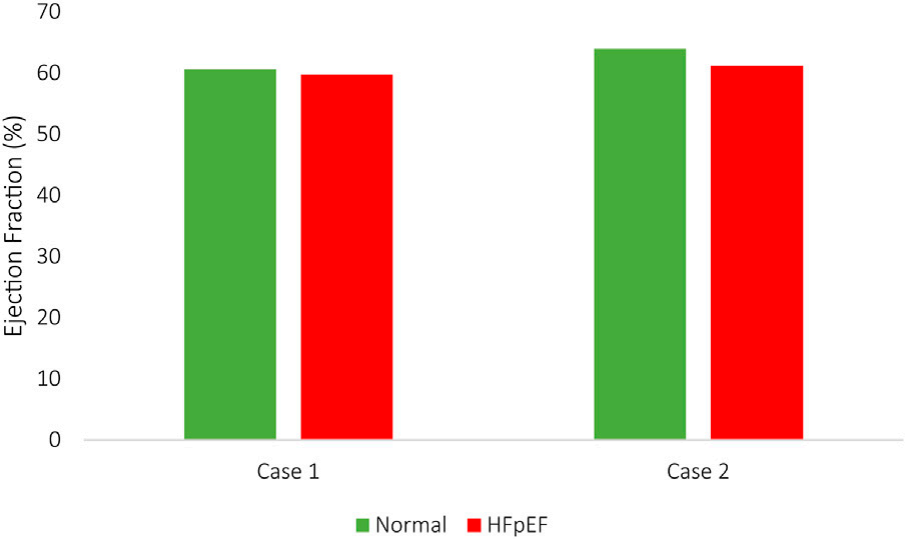
The ejection fraction of normal and HFpEF configurations.

The EDV and ESV compared adequately between the imaging studies and the computational results, with less than a 5% inaccuracy. Accordingly, *in silico* stroke volume (SV) matched *in vivo* measurements for all HF models in all configurations. The computed SVs in Case 1 were 32.2 ml and 31.2 ml for normal and HFpEF conditions, respectively, compared to 33.5 ml and 30.5 ml *in vivo* measurements. Similarly, in Case 2, the SVs were 35.4 ml and 34.0 ml, vs. 36.1 ml and 34.4 ml measured *in vivo*. The changes in EF in both HF conditions are illustrated in Figure 7.

### Strain assessment and validation

Supplementary Video S1 shows the beating heart models during the cardiac cycle with varying maximal principal strains. The HFpEF models for Case 1, with an increased hypertrophy, have shown similar magnitudes relative to the corresponding normal heart, whereas the strains in the HFpEF model for Case 2 have shown a reduction. These strains were computed per element and may suggest the focused impact of hypertrophy on specific segments. To further assess the strain and to validate the results, global FE strain measurements in the longitudinal and circumferential directions were computed across the endocardium and compared to *in vivo* strain data. Although echo-derived strain data is considered reliable, it is most challenging given the time-dependent variation in position and particularly while analyzing cross-sectional images. The end-systolic phase was chosen as the target configuration, and the end-diastolic phase was defined as the reference configuration. To best fit the FE results to the *in vivo* strain measurements, curve lengths from long and short axis slices were measured manually rather than using the built-in strain results.

The results of the FE models have shown a reduction in myofiber longitudinal strain within the LV, with 18% vs. 15% for Case 1 and 17% vs. 14% for Case 2. A slight increase was observed in the endocardial circumferential strain: 21% vs. 23% and 25% vs. 26% for Cases 1 and 2, respectively. The global strains, which were calculated from the FE results, are commensurate with the clinical measurements listed in Table 6. The accuracy in predicting longitudinal strain was higher than the circumferential strain. The latter is more complicated to accurately measure given the image resolution and the high variability between slices. Furthermore, the overall measurements can also be affected by the myofiber orientation.

**TABLE 6:** Endocardial longitudinal and circumferential strains in percentages from manual echocardiogram length measurements.

	Case 1		Case 2	
	Longitudinal	circumferential	Longitudinal	circumferential
Normal	20%	28%	27%	30%
HFpEF	12%	28%	18%	32%

## Discussion

In this study, we used cMRI scans from HFpEF induced pigs to determine changes in material properties and to assess different phenotypes of the disease. Subject-specific FE models were developed based on the living heart porcine model to represent anatomies before and after induction.

### Geometrical reconstruction

The geometries in this study were constructed to match the cMRI recordings by morphing the cardiac porcine model of the living heart. It is considered a highly demanding model, as it enables the investigation of the four heart chambers as well as the coupled electrophysiology. The use of animal subjects and a non-symmetrical geometry enables realistic modeling of the disease and allows exploring alterations in the material properties more precisely.

The phenotype classification was determined according to the degree of hypertrophy in response to the increased overload. This compensatory mechanism can result in heart failure when it occurs excessively (Kehat and Molkentin, 2010). The computed LVMI has shown a substantial twofold increase in myocardial mass in both pigs. In Case 1, myocardial thickening was greater, implying a more pathologic scenario.

### Material properties

All FE models were calibrated from the same initial volume to normalize them and to reduce material discrepancies due to volume alterations (Figures 4, 5). Furthermore, selecting a reference point allowed to limit the number of solutions of the multivariate analysis, as there is often more than one possible solution. For each model, the best solution was chosen after running the stochastic algorithm three times for each scenario, using the same scoring method. The set of material properties with the optimal score was used for our comparison.

The pressure-volume curves of both cases before the induction of HFpEF are in close range, attesting to the resemblance between the two initial calibrated configurations. The volume was estimated from a small number of slices, resulting in a measurement error range of 10% (El-Rewaidy and Fahmy, 2016), which is more than the volumetric calibration error. Differences in pressure magnitudes of less than 1 mmHg are also considered minor (Tolia et al., 2018), since the physiologic pressures were estimated from E/e’ measurements according to Nagueh’s formula (Nagueh et al., 1997).

It is important to underscore that the curves are ultimately determined by the patient-specific anatomy and the material properties, as demonstrated by the variability between the post induction configurations and their corresponding curves. To eliminate alterations in the cardiac anatomy due to remodeling, thus isolating the impact of the passive material parameters, the initial anatomy was modified to fit the HFpEF pressure volume curve (Figure 7). The change was primarily isotropic (*a*, *b*), with negligible impact on the remaining parameters. These results are not biased by initial parameter constraints (Nikou et al., 2015), nor by parameter significance ranking (Nair et al., 2007). Importantly, to keep the parameters in the physiological range, the stiffness of the heart had to rise, resulting in a drop in the incompressibility parameter *D*, which yielded an increase in the bulk modulus of three orders of magnitude greater than the increase in parameter *a* (Göktepe et al., 2011). The reduction in the incompressibility parameter *D* also leads to a shift of the Klotz curve toward lower volumes (to the left). The range of the *D* parameter (0.1–0.25 MPa) was chosen to ensure that the influence of incompressibility on the material properties is minimal (Sack et al., 2018). Interestingly, the change in the myocardial passive behavior is predominantly isotropic direction 2) when comparing the same geometry before and after induction (Table 5). Our calibration algorithm ran under identical conditions in all models, including initial volume setting as well as predefined boundaries for each parameter, to assure normalization and to reduce material discrepancies due to volume alteration. No additional constraints were added, allowing all the parameters to change randomly until an optimal solution was obtained. Importantly, this is the first article to compare HFpEF to normal heart of the exact subject, thus allowing for an accurate assessment of myocardial changes. Our findings suggest that cardiac hypertrophy may enable the preservation of the passive material properties in the physiological range while tissue incompressibility is maintained. It is consistent with the convention that hypertrophy serves as an initial compensatory response to sustain cardiac function (Hein et al., 2003). Finally, we compared two HFpEF phenotypes. High variability in material properties was observed following the realistic calibration of the two models (Table 4). The hypertrophy magnitude triggered dissimilar changes in different parameters, emphasizing the importance of addressing all material properties for accurate calibration.

### Cardiac cycle simulation

Pressure-volume curves of the LV (PV-loops) were generated to represent normal and HFpEF conditions (Figure 6). The converged cardiac cycles accurately described clinical observations, with a difference of less than 5%. This error is minor, considering physiological variations in volume, pressure, and timing during cardiac function and over *in-vivo* sampling. While the PV-loops representing the normal configurations of the two cases are in close resemblance, this is not the case in the HFpEF configurations. The differences in the HFpEF curves are demonstrated both in the PV values and the shape of the curves. These changes can be explained by the degree and direction of the hypertrophy, which engenders alterations in the material properties.

A quantitative assessment of the models was performed by comparing the strains of the HFpEF and normal configurations. The strain measurement difference between the cardiac subjects before and after induction has shown an expected reduction in longitudinal strain at diastole. The FE estimations agreed with the clinical data, with only a negligible increase in the circumferential strain. The relative error between the FE results and the clinical data can be explained by the different imaging modalities, i.e., cMRI vs. echocardiography, respectively. Furthermore, the model geometries were reconstructed without adjusting the orientation of the muscle fibers. Nonetheless, the trends were largely the same, and the cardiac behavior was similar.

### Study limitations and future directions

Several key factors contribute to diastolic HF formation and include both systolic and diastolic abnormalities (Sengupta and Marwick, 2018). The FE analysis in this study addressed the alteration in mechanical properties of the LV as well as geometrical changes such as hypertrophy (Aurigemma and Gaasch, 2004; Torres et al., 2020). Investigation of the LV systolic mechanics is beyond the scope of this paper. In our investigation, we assumed the change in material properties to be homogeneous across the LV and uniform in all directions. The calibration process was based on echocardiographic estimation of intra-cardiac pressures normalized by weight rather than direct catheter readings. Moreover, the FE models relied on cMRI recordings that were only obtained before and after HF induction. Sequential MRI monitoring during the induction process could provide additional information on disease progression and tissue remodeling and is the goal of our subsequent study. Here, we sought to investigate changes in global passive material properties of similar baseline anatomies to induce different representations of HFpEF.

## Conclusion

This study evaluates material property alteration in two different phenotypes of HFpEF using subject-specific cardiac models for porcine hearts before and after the application of progressive and prolonged pressure overload. The change in the myocardial passive behavior was isotropic and its magnitude was heavily reliant on the degree of hypertrophy. When hypertrophy was excluded, elevation in incompressibility was enforced, triggering alterations only in parameter *b*. This study is the first to quantify and compare alterations in material properties and incompressibility in different HFpEF phenotypes. The results underline the advantage of computational modeling in understanding complex cardiac representation *via* tissue behavior quantification.

## Data Availability

The original contributions presented in the study are included in the article/Supplementary Material, further inquiries can be directed to the corresponding author.
