# Supplementary material for: Material property alterations for phenotypes of heart failure with preserved ejection fraction: A numerical study of subject-specific porcine models
Source: Front Bioeng Biotechnol. 2022 Oct 14;10:1032034. doi: 10.3389/fbioe.2022.1032034 (PMC9614036; doi:10.3389/fbioe.2022.1032034)
Supplement: Supplementary file 1 [file DataSheet1.pdf]

Supplementary Material

Material property alterations for HFpEF phenotypes: A numerical study of subject-specific porcine models

Jonathan Weissmann,[1] Christopher J. Charles,[2,3,4] A. Mark Richards,[3,4] Choon Hwai Yap,[5] and Gil Marom[6]

[1] Department of Biomedical Engineering, Tel Aviv University, Israel

[2] Department of Surgery, Yong Loo Lin School of Medicine, National University of Singapore, Singapore

[3] Cardiovascular Research Institute, National University of Singapore, Singapore

[4] Christchurch Heart Institute, Department of Medicine, University of Otago, Christchurch, New Zealand

[5] Department of Bioengineering, Imperial College London, UK

[6] School of Mechanical Engineering, Tel Aviv University, Israel

Corresponding Author: Gil Marom, Ph.D.

Address: School of Mechanical Engineering
Tel Aviv University
Tel Aviv 6997801, Israel

Telephone Number: (972) 3-640-8788

E-mail: maromgil@tau.ac.il

1. Supplementary Tables and Videos

Supplementary Video S1: Maximum principal strain (%) distribution in the four models throughout the last cardiac

Table S1: Comparison of the circulatory system parameters.

	Case 1		***Case 2***	
	**Normal**	**HFpEF**	**Normal**	**HFpEF**
Aortic valve resistance	1	4	2	3
Systemic resistance	8	160	18	160
Venous resistance	132	100	75	55

2. Additional details for "Klotz" curve configuration

The "Klotz" curve can be configured using a single known scaling point to analytically predict the end-diastolic pressure-volume relations, as suggested in the original paper of Klotz et al. (2006). Volumes at the pressure of 0 mmHg (V_0) and 30 mmHg (V_{30}), are computed from the scaling point:

$$V_0 = V_m(0.6 - 0.006P_m) \qquad ; \qquad V_{30} = V_0 + \frac{V_m - V_0}{0.302P_m^{0.358}}$$

where V_m, P_m are the volume and pressure of the scaling point. The resultant V_{30} can then be used to calculate the coefficients α and β:

$$\alpha = 30/V_{30}^{\beta} \qquad ; \qquad \beta = \frac{\log{(P_m/30)}}{\log{(V_m/V_{30})}}$$

Finally, the entire end-diastolic pressure volume relation can be expressed as $P = \alpha V^{\beta}$.

3. Additional details for active contraction

The constitutive equations of the active stress are (Guccione et al., 2001):

$$\sigma_{af(t,E_{ff})} = \frac{T_{max}}{2}\frac{Ca_0^2}{Ca_0^2 + ECa_{50}^2(E_{ff})}\left(1 - \cos(\omega(t, E_{ff}))\right)$$

$$ECa_{50}^2 = \frac{(Ca_0)_{max}}{\sqrt{\exp{[(B(l - l_0)] - 1}}} \qquad ; \qquad l = l_R\sqrt{2E_{ff} + 1}$$

$$\omega = \begin{cases} \pi \frac{t}{t_0} & \text{when} \quad 0 \le t \le t_0 \\ \pi \frac{t - t_0 + t_r}{t_r} & \text{when} \quad t_0 \le t \le t_0 + t_r \\ 0 & \text{when} \quad t \ge t_0 + t_r \end{cases} \quad ; \qquad t_r = ml + b$$

where T_{max} is the isometric tension of the largest sarcomere length with the highest calcium concentration multiplied by a calcium concentration determining term and a contraction timing governing term, both of which are dependent on sarcomere length l. The timing of contractile function corresponds with the heart rate and timing of the cardiac cycle, which is imposed by a time (t) dependent modulus function.

For calibration purposes, a consistent alteration in contractility was facilitated by altering the T_{max}, changing simultaneously both the total contractile force of the tissue as well as the aforementioned parameters related to active behavior.

The model was initialized with the passive material properties previously calibrated. Then, the active calibration followed methods from previous studies (Genet et al., 2014; Guccione and McCulloch, 1993; Sack et al., 2016). The pressure in the LV was raised to EDP, increasing the LV volume more than the EDV that was used for the calibration of the passive calibration. Next, the active tension was elevated to enforce systolic contraction and thereby reducing LV volume. The T_{max}, which governs the systolic contraction, was scaled iteratively using a gradient descent algorithm to match the end-systolic volume obtained from the simulation to the animal study measurement.

Supplementary references

Genet, M., Lee, L.C., Nguyen, R., Haraldsson, H., Acevedo-Bolton, G., Zhang, Z., Ge, L., Ordovas, K., Kozerke, S., Guccione, J.M., 2014. Distribution of normal human left ventricular myofiber stress at end diastole and end systole: a target for in silico design of heart failure treatments. J. Appl. Physiol. 117, 142–152. https://doi.org/10.1152/JAPPLPHYSIOL.00255.2014

Guccione, J.M., McCulloch, A.D., 1993. Mechanics of active contraction in cardiac muscle: Part I--Constitutive relations for fiber stress that describe deactivation. J. Biomech. Eng. 115, 72–81. https://doi.org/10.1115/1.2895473

Guccione, J.M., Moonly, S.M., Moustakidis, P., Costa, K.D., Moulton, M.J., Ratcliffe, M.B.,

Pasque, M.K., 2001. Mechanism underlying mechanical dysfunction in the border zone of left ventricular aneurysm: A finite element model study. Ann. Thorac. Surg. 71, 654–662. https://doi.org/10.1016/S0003-4975(00)02338-9

Klotz, S., Hay, I., Dickstein, M.L., Yi, G.H., Wang, J., Maurer, M.S., Kass, D.A., Burkhoff, D., 2006. Single-beat estimation of end-diastolic pressure-volume relationship: a novel method with potential for noninvasive application. Am. J. Physiol. Heart Circ. Physiol. 291. https://doi.org/10.1152/AJPHEART.01240.2005

Sack, K.L., Baillargeon, B., Acevedo-Bolton, G., Genet, M., Rebelo, N., Kuhl, E., Klein, L., Weiselthaler, G.M., Burkhoff, D., Franz, T., Guccione, J.M., 2016. Partial LVAD restores ventricular outputs and normalizes LV but not RV stress distributions in the acutely failing heart in silico. Int. J. Artif. Organs 39, 421–430. https://doi.org/10.5301/IJAO.5000520
